# Incidence of fall accident in Suseong district of Daegu Metropolitan City, Republic of Korea

**Published:** 2019-08

**Authors:** Jeongyee Bae, Sang-Hyun Noh, Sang-Hun Kim, Hong-Sun Song, Chang-Hyo Bae, Dong-su Han, Hyewon Lee

**Affiliations:** ^*a*^Chair, Inje University Institute for International Safe Promotion and Professor, Inje University, Korea.; ^*b*^Public Official, Suseong District of Daegu Metropolitan City, Republic of Korea, Korea.; ^*c*^Research Assistant, Inje University Institute for International Safe Promotion, Korea.

## Abstract

**Background:**

Among the various types of injuries in the world, falls are one of the most important health problem related issues, especially for modern societies with aged populations. Korea, a country where industrialization and urbanization have made building structures and living spaces complex, has also been facing the emerging danger of falls. So, among the causes of death due to unintended injury accidents, falls are second reason of injury worldwide following traffic accidents. Falls are important causes in increase of morbidity rates, hospitalization rates, death rates and medical fees. Falls are not unfortunate accidents rather, they are preventable and predictable. Therefore, we needs to identify the incidence and causes of falls. Objectives The purposes of this study are to identify the incidence of fall accident in Suseong district of Daegu metropolitan city and to develop the evidence based fall accident program for Suseong district.

**Methods:**

In order to identify the incidence of falls of the citizens of Suseong district, authors analyzed the annual report on the causes of death statistics from the National Statistical Office and the injury data from Fire Station 119 Rescue Activity Daily Record. As well, in order to understand the current state of falls in households, an epidemiological survey was conducted to people from 1,977 households in Suseong district by home visited. The collected data were analyzed using SPSS WIN 17.0 based on the research objectives, and the frequency and percentages were investigated.

**Results:**

According to the data from the National Statistical Office, the death rate due to falls was 4.1 people (per 100,000 persons). In terms of age, elders aged 65 and over was the highest death rate with 17.0 out of 100,000 persons. In the injury transfer rate analyzed from rescue records of 119, accidents due to falls were highest with 2.3 cases out of 1,000 persons. In terms of age, elders aged 65 and over was the highest injury rate with 9.2 out of 1000 persons. After analyzing the injury causes of the citizens of Suseong district through household visits, the highest rate of falls was 43.7%, followed by traffic accidents (24.9%) and bump injuries (21.1%). Therefore, we were able to confirm once again that the fall prevention program is a very important program for improving the safety of citizens. Housework (29.4%) was the most common cause of falls. The most common places for falls were living rooms (22.6%), followed by restrooms and entrance.

**Conclusions:**

Mortality due to falls can be reduced through education, environmental improvements, and safety policies. It is necessary to conduct preventive education for elderly people who are vulnerable to falls, and to apply gymnastic exercises that can enhance balance and lower extremity muscle strength. In addition to, it is necessary to strengthen regulations on safety facilities at the senior community center. Therefore the government and related organizations should put fall prevention as the primary task and spread an effective evidence-based fall prevention program based on result of research.

**Keywords:**

Fall, Injury, Safety, Safety promotion

